# Emotional Androgyny: A Preventive Factor of Psychosocial Risks at Work?

**DOI:** 10.3389/fpsyg.2018.02144

**Published:** 2018-11-26

**Authors:** Leire Gartzia, Jon Pizarro, Josune Baniandres

**Affiliations:** ^1^Department of People Management in Organizations, University of Deusto, Bilbao, Spain; ^2^Department of People Management in Organizations, Deusto Business School, Bilbao, Spain

**Keywords:** gender, psychosocial risks, androgyny, emotional competences, work

## Abstract

Although previous studies have acknowledged the connections between gender and emotional competences, more research is needed on how gender and emotion interact to influence psychosocial risks at work. This paper addresses how gender stereotypes and emotions simultaneously act as psychosocial antecedents of organizational stress. Following the principles of psychological androgyny, we propose that a combination of communion and agency can serve as a preventive factor at work and lead to healthier responses by providing a wider range of emotional competences to deal with organizational demands. Following previous methodological approaches, we include a quantitative review about scientific research on occupational health in the PsycINFO database during the period 1980–2017 from a multidimensional gender perspective that differentiates between studies addressing the topic from either sex, gender or gender identity dimensions. Finally, we propose new analytical directions to deal with psychosocial hazards at work by underscoring some of the complex ways in which gender and emotional competences influence psychosocial risks at work.

## Emotional Androgyny: A Preventive Factor of Psychosocial Risks at Work?

Every-day work experiences with potentially major psychological consequences for employees depend on individual characteristics and workplace features that include physical, emotional, and social work (Patterson et al., 1997; Grandey, 2000). At the organizational level, many workplace characteristics can be a critical source of stress, ranging from work design and practices associated with ergonomics (Hoke, 1997; Patterson et al., 1997) to organizational climate (Bond et al., 2010), leader–employee relationships (Blanchard, 1993; Cooper and Cartwright, 1994; Hornstein, 1996), or supervisor styles and support (Babin and Boles, 1996). Employees individual characteristics and competences are also important because the experience of stress is dependent on individuals’ abilities to cope with such demands placed on them by their work (Coté, 2005; Hackney and Perrewé, 2018). Thus, emotion regulation strategies are critical to how employees respond to work stressors (see Coté, 2005) and emotion regulation is an important predictor of job strain (Ashforth and Humphrey, 1993; Grandey and Brauburger, 2002; Pugh, 2002).

Although researchers have acknowledged the relevance of emotion regulation strategies on how employees respond to work stressors, they have only begun to raise concerns about their gendered nature. This approach is important because the experience, expression and regulation of emotions as well as expectations about men’s and women’s functions are deeply gendered (Frijda and Mesquita, 1994; Brody, 1997; Bindu and Thomas, 2006; Gartzia et al., 2012b). Consistent with gender stereotypes that prevail in Western societies, emotion expression and management is largely gendered (Brody and Hall, 2000; Fischer and Manstead, 2000). Women are socialized to be communal, with stronger emphasis on other-oriented emotional dimensions related to showing interest in emotions, being sensitive to what others feel, or expressing feelings. In contrast, men are socialized to be agentic, which implies more stress on self-confidence, strength, and assertiveness (Parsons et al., 1955; Stewart and McDermott, 2004). Despite the relevance of a critical examination of psychosocial risks and emotional competences from a gender perspective, examinations of whether and how emotional and psychosocial responses at work are gendered are rare (see Cifre et al., 2013, 2015).

To respond to this gap, the current article acknowledges the gendered nature of psychosocial risks at work and provides theoretical background to understand both workplace characteristics and subsequent employees’ emotional responses from a gender perspective, which can shed light on the relevant issue of how male and female employees can potentially better respond to stressors at work. We first introduce the relevance of emotion and emotional competences for employees’ psychological responses and work stress and outline the gendered nature of emotion. Second, we describe the most common psychosocial risks at work from a gender perspective, including a quantitative review about scientific research in the field during the period 1980–2017. To do so, we adopt an approach that explicitly differentiates between sex and gender dimensions, with particular emphasis on studies about gender identity. Finally, we adopt a social identity perspective that underscores the interactive roles of gender identity and emotional competences in reducing psychosocial risks at work. We discuss the potential application of “emotional androgyny” to deal with occupational hazards, suggesting that a theoretical and empirical approach based on the promotion of counter-stereotypical gender competences can be a particularly useful approach to understand psychosocial risks in organizational settings.

## Work and Stress From the Perspective of Emotion

Work is a critical source of stress (Ganster and Schaubroeck, 1991), with more than 40 million people suffering work-related stress across the EU (Paoli and Merllié, 2001). As a widespread occurrence, work stress is of the most common sources of disease at work with estimated annual losses of around €20 billion (European Foundation for the Improvement of Living and Working Conditions, 2007; Milczarek et al., 2009). Consistent with the relevance of the topic, the issue of how employees respond to workplace events from the perspective of mental health and psychological responses has become a relevant theme for practitioners (cf. King, 1995; Neville, 1998; Rajgopal, 2010) and the media (cf. Coleman, 1997). Likewise, academic research on work-related stress is growing rapidly (cf. Warr, 1990; Briner, 1994; Cooper and Cartwright, 1994; Smith et al., 1995; Mark, 2008) with a particular focus on understanding explanatory mechanisms and identifying factors that may help employees overcome negative work experiences.

The critical relevance of stressful workplace events and subsequent affective experiences is captured in the foundations of Affective Events Theory (Weiss and Cropanzano, 1996), which addresses the causes and consequences of employees’ work experiences and how they are inherently linked to emotions and moods. Emotions represent physiological states of arousal that provide cues about the environment and get individuals in a physical state to respond to the situation (Frijda, 1986). These tendencies to respond to environmental experiences producing emotion are based on primitive emotional tendencies (e.g., attack or escape) that occur in subtle ways in today’s society and organizations. As proposed by general theories of emotion and stress (Lazarus, 1999), the experience of stress captures unfavorable person–environment relationships in which individuals alter their interpretations of such relationships to make them appear emotionally more favorable. Such an effort – called coping – also allows that people do not react inappropriately in terms of social behavior (Lazarus, 1991; Grandey, 2000).

As proposed by Affective Events Theory (Weiss and Cropanzano, 1996), affective experiences of employees at work are particularly relevant and have consequences at many dimensions of organizational behavior and health. Thus, underlying mechanisms associated with emotions and emotional competences are critical in understanding how work experiences influence psychosocial risks at work. Emotion regulation strategies are particularly relevant to how employees respond to work stressors (see Coté, 2005) and emotion regulation is an important predictor of strain (Ashforth and Humphrey, 1993; Grandey and Brauburger, 2002; Pugh, 2002). Generally speaking, emotion regulation refers to efforts to increase, maintain, or decrease certain components of emotion (Gross, 1999). Because the experience and expression of emotion is both intrapersonal (i.e., with effects of emotions on individuals’ own behavior) and interpersonal (i.e., with effects of emotions on the behavior of other individuals; Frijda and Mesquita, 1994; Keltner and Kring, 1998; Morris and Keltner, 2000), emotional responses and emotion regulation strategies are also relevant mechanisms in how work experiences result in negative psychosocial outcomes like burnout, job dissatisfaction, or stress at different levels (see Grandey, 2000).

A growing number of studies based on the notion of emotional intelligence have also analyzed how understanding and regulating one’s emotions and those of others promote emotional and intellectual growth and can have key effects on physical as well as psychological health (Mayer et al., 2008). Emotional intelligence is generally defined as the ability to pay attention, understand, and regulate emotions (Mayer et al., 2008). Research that analyzes this type of intelligence has grown substantially and has shown that it is an important predictor of variables such as satisfaction with life and the quality of interpersonal relationships (e.g., Ciarrochi et al., 2000), as well as psychological adjustment and reduction of work stress (e.g., Bar-On et al., 2000). The positive effect of emotional intelligence competences has been revealed not at the subjective-experiential level, but also with neuroendocrine correlates (Mikolajczak et al., 2007).

The relevance of emotional competences to overcome stress is clearly understood from the perspective of individual competences of employees (i.e., considering emotional competences as a relevant individual resource to better cope with environmental demands at work), but note that emotional competences are also a relevant buffer of psychosocial risks from the perspective of the organization (e.g., leadership styles, cultural norms, and shared values). The social learning theory of aggression postulates that reducing negative behaviors in an environment through reinforcement and modeling can reduce such abusive behavior by showing individuals that aggressive behaviors are not welcome in a social context (Bandura, 1983; Hackney and Perrewé, 2018). Thus, the rationale that emotional competences can be a preventive factor of psychosocial risk is fundamentally the same from the perspective of the “receiver” (e.g., an employee experiencing stress due to an abusive supervisor) and the “source” (e.g., the supervisor herself/himself displaying hostile behaviors directed toward the employee). From this perspective, it is understood that workplace structures would also benefit from self-regulation and awareness processes directed at reducing negative behaviors, values, and implicit norms.

Extending this viewpoint, Hackney and Perrewé (2018) developed a process model to understand antecedents and consequences of behaviors with potentially negative psychosocial effects at work (i.e., with a particular focus on workplace abuse). They argued that organizational factors (e.g., strong cultural norms supporting aggression) create contexts in which individual negative behavior is reinforced in subtle ways (e.g., by for instance allowing harassment of minorities and aggressive individual behavior but “pretending it was just a joke”; Hackney and Perrewé, 2018, p. 76). These environmental factors combine with the individual attitudes and emotional competences of people in powerful positions at work (i.e., leaders). Thus, leaders’ ability to control themselves and use self-regulatory resources to resist temptations such as yelling to coworkers or making aggressive jokes is critical (Hackney and Perrewé, 2018).

## The Gendered Nature of Emotional Competences

Consistent with the idea that women are more emotional than men and the thought that men and women are emotionally different (Brody and Hall, 2000), sex differences have traditionally emerged in emotional competences and responses (Hopkins and Bilimoria, 2008). For instance, emotion research has shown that women generally outperform men on tests about the ability to accurately decode others’ emotion displays (see Hall et al., 1978). Women are socialized with communal traits that involve relational emotional competences associated with showing interest in emotions, being sensitive to what others feel, or expressing feelings. Men, in contrast, are socialized with agentic traits that involve stronger emphasis on self-confidence, strength, and assertiveness (Shields, 2002; Stewart and McDermott, 2004). Likewise, women are socialized to experience guilt, shame, and depression (Allen and Haccoun, 1976; Brody, 1997), higher levels of sense of failure and sadness (Oliver and Toner, 1990), intense fear (Speltz and Bernstein, 1976; Cornelius and Averill, 1980), and rumination on negative emotions (Nolen-Hoeksema, 1987).

Stemming from the large body of research analyzing gender and emotion, the possibility that women are more emotionally intelligent than men has also attracted attention among researchers. This supposed greater ability of women to be emotionally intelligent is in line with the widespread and uncontested idea that women are more “emotional” than men, as well as with previous studies showing that women have higher interpersonal sensitivity to verbal and non-verbal cues, are better at understanding emotional information and tend to empathize more than men (Lloyd, 1984; Tannen, 1990; Baron-Cohen, 2002; Shields, 2002; Brody and Hall, 2008). Not surprisingly, meta-analytical studies have shown that women are overall more emotionally intelligent than men (see Joseph and Newman, 2010). Acknowledging the complexity within the general umbrella of emotional intelligence, studies have also underscored the “masculine” nature of emotional dimensions with an agentic content, such as regulation of negative emotions in a way that is constructive to the self (see Gartzia and López-Zafra, 2016). Accordingly, men are often better than women at handling negative emotions and having an optimistic outlook (Livingstone and Day, 2005; Gartzia and van Engen, 2012).

Given stereotypical connections about femininity and emotionality, rules about the emotions that employees have to display at work are often gendered too. Likewise, although work-related stress is not limited to certain job positions, research has suggested that occupations such as those of social work – i.e., a stereotypically feminine occupation – represent particularly stressful work contexts (Lloyd et al., 2002). Because social work generally requires interpersonal interactions whereby emotional labor is particularly salient, social work is often seen as an inherently stressful occupation. An additional issue to consider is that social workers – generally women – often have an inherent disposition and motive in their choice of profession to be oriented to people and be helpful, which can be a particularly relevant contributor to stress (Borland, 1981; Egan, 1993; Acker, 1999).

The gendered nature of emotion is also manifest in managerial positions. For instance, research has shown that abusive behavior occurs when supervisors are not able to self-regulate aggressive behaviors such as expressing anger and yelling to employees (Baumeister, 2002; Mawritz et al., 2017). Because anger signals dominance and power, it is more closely associated with masculine roles and therefore male targets expressing anger are more often positively valued than female targets expressing such emotion (Brody and Hall, 2000; Shields, 2002). However, leaders who feel and express other emotions such as excitement and energize themselves are likely to similarly energize their followers, whereas leaders who feel negative emotions such as distress and do not regulate them are likely to similarly activate their followers in a negative way (George et al., unpublished). These asymmetries can also have important implications for psychosocial risks at work.

All in all, these asymmetries generate differences in the expectations and emotional resources of men and women to respond to environmental factors. Because emotional competences are key components to deal with workplace stressors and reduce stress (Ashforth and Humphrey, 1993; Grandey and Brauburger, 2002; Pugh, 2002; Coté, 2005), a critical examination of emotional competences from a gender perspective can shed light on the relevant issue of how – male and female – employees respond to psychosocial risks at work. However, general studies analyzing emotions have often been limited to the variable of sex as the main predictor of individual differences (see, for instance, Baron-Cohen, 2003). Importantly, the assumption that sex differences in emotionality prevail has primarily constituted an implicit assumption that has yet to be subjected to further exploration (Conway, 2001; Wester et al., 2002). Indeed, critical reviews of sex differences in emotions have shown that differences between men and women in emotionality are small, inconsistent, and context-dependent (see Ickes et al., 2000; Wester et al., 2002), suggesting that emotional dimensions are influenced by gender stereotypes and roles in complex ways.

Acknowledging these complexities, an increasing number of studies have called for the need to consider the role of communal and agentic traits when explaining the connections between gender and emotional competences. Because sex differences in emotional intelligence are due at least in part to identification with different gendered traits (Gartzia and van Engen, 2012; Gartzia et al., 2012a), examining within-sex gendered differences is also important to fully understand how men and women acquire different emotional intelligence profiles. This perspective is coherent with studies showing the incremental validity of gender identity traits over sex predicting emotional competences (i.e., women’s higher scores in emotional attention and expression and men’s higher scores in emotional repair; Guastello and Guastello, 2003; Gartzia et al., 2012a). Thus, a social identity approach can help underscore how going beyond gender stereotypes and incorporating counter-stereotypical identity traits into the self can improve subsequent emotional responses in the workplace, thereby providing a context to better overcome psychological hazards.

## Occupational Risks From a Gender Perspective

Given the connections between emotions and psychosocial risks at work, understanding the gendered nature of emotions can inform occupational health researchers. However, because psychosocial risks at work can derive from a wide variety of sources, their associations with gender and emotional responses are also difficult to simplify. Men and women are socialized with different traits and expectations about their functions in social and organizational roles, resulting in fundamental differences in how they experience organizational life in relation to a variety of domains including promotion opportunities, salary, interpersonal relationships, number and distribution of working hours, or leadership styles (see Eagly et al., 2012; Hausmann et al., 2014). Likewise, men have compared with women higher career-related expectations of workplace commitment in terms of paid work hours, which are subsequently associated with better self-evaluations and emotional well-being (Gartzia et al., 2012b). Some of the effects associated with these differences are that women experience more emotional exhaustion at work, whereas men experience other symptoms like depersonalization (Purvanova and Muros, 2010).

Despite progress in gender equality at work, there is also a substantial gender gap in the domestic division of labor (Kan et al., 2011; Hausmann et al., 2014). With current organizations’ work intensity (Chirico, 2017), a particularly common source of stress at work is the work–family conflict, namely a “form of interrole conflict in which the role pressures from the work and family domains are mutually incompatible in some respect” (Greenhaus and Beutell, 1985). Results about sex differences in psychological reactions to work–life balance such as the work–family conflict, however, are inconclusive and the connections between gender and work–life balance are still unclear (e.g., Frone, 2003; Eby et al., 2005; Korabik et al., 2008). Because women often prioritize domestic interests by for instance taking maternity leaves or reducing their number of working hours (Hausmann et al., 2014), their career penalties due to domestic responsibilities can clearly represent an occupational risk factor associated with expectations about their feminine gender role. Indeed, the gender literature has repeatedly shown that women’s greater assumption of domestic work is one of the most important factors for the persistence of gender discrimination and result in career penalties that can be very negative for women’s advancement and status at work (Bittman et al., 2007; Eagly et al., 2012; European Commission, 2015). These asymmetries are likely to influence work-related stress given the generalized assumption that employees should prioritize the interests of the organization over personal interests as a signal of commitment (Powell, 1990).

Additional evidence about the influence of gender in how individuals are confronted with and respond to psychosocial risks derives from the rich literature on leadership and management. There is accumulated confirmation that prototypes about leadership effectiveness are consistent with a stereotypically masculine ideal whereby agentic traits (e.g., competence and self-decision) prevail over communal and emotion-related features such as being sensitive to others’ emotions (see Koenig et al., 2011). Thus, despite the growing interest on emotions at work (Brief and Weiss, 2002), the myth of rationality is deeply rooted in political and social organizations, and it is related to stereotypical masculinity and a belief in objectivity and reason (Collinson and Hearn, 1996; Fineman, 2010). As a consequence, socioemotional skills are often undervalued in organizations and particularly in areas of power where a *Think manager-Think male* stereotype prevails (Schein et al., 1996). The pervasiveness of this stereotype can be very important in the organizational practice because leaders’ emotional responses and abilities to understand and regulate emotions are associated with a wide range of well-being outcomes and emotional responses from employees (see Avolio and Gardner, 2005; Bono and Ilies, 2006).

Taken together, these findings reveal the complexities implicit in how gender roles and expectations operate in organizations and result in psychological reactions for male and female employees. These complexities are only partially captured through the work and stress literature and the many inconclusive findings in relation to the effects of gender on the different forms of psychosocial risks at work. Implicit in our approach about the relevance of a critical gender perspective is the idea that to better understand these complexities, it is important to examine the different components through which the sex and gender dimensions differently operate in shaping employees’ responses at work. Following previous research (e.g., Eagly et al., 2012), we use the term sex to denote the grouping of people into female and male categories and the term gender to capture the meanings that societies and individuals ascribe. In the next section, we provide a quantitative review about the prevalence of a sex vs. a gender perspective in the workplace literature.

## Analytical Section: Number and Types of Gender Articles in the Literature

**FIGURE 1:**
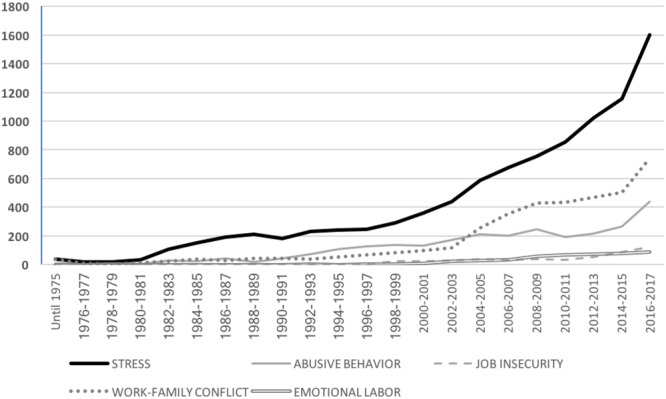
Total number of articles in the most common psychosocial risk categories.

Following previous methodological approaches of addressing the state in the art in a given area of gender research by counting relevant papers in the previous literature (Eagly et al., 2012; Gartzia and López-Zafra, 2014, 2016), we examined research in several psychosocial domains during the 1980–2017 period following the method of counting relevant journal articles from the PsycINFO database. Note that 2017 is the most recent date for which complete data are available in the database as of this writing. To assist this analysis, quantitative data about the growth of scientific research papers in relation to the most commonly studied psychosocial risks is provided. In particular, we included a review of the five psychosocial risks at work that have been shown to represent the most commonly studied dimensions of work-related stress in the literature and by public institutions in charge of safety at work in Europe (Paoli and Merllié, 2001; Leka et al., 2008): stress, abusive behavior, job insecurity, emotional labor, and the work–family conflict (see specific keywords below). As shown in Figure 1, the number of research articles about workplace stress in the period 1980–2017 (i.e., 9,805 articles) is substantially higher than the overall number of articles about other risks (4,084 articles in the work–family conflict category; 2,842 in the abusive behavior category; 536 articles in the job insecurity category; and 505 articles in the emotional labor category). These results point to the particular relevance of workplace stress and the work–family conflict in the occupational risk literature. However, this pattern of results varies when examined from a gender perspective, as explained below.

To accurately understand how psychosocial risks are linked to gender dimensions in previous research, we deconstructed the associations between gender and psychosocial risks into their component parts. One issue is whether psychosocial risks differently influence women and men at work, either generally or in relation to some particular stressors. Following previous research (e.g., Eagly et al., 2012, 2014) using the term sex to denote the grouping of people into female and male categories, we included a category of studies explicitly examining sex differences and similarities in each psychosocial risk (i.e., “*sex difference^∗^*” *OR* “*sexual development*” *OR* “*sex hormones*”). Following Hyde (2005), we refer to this perspective as a “sex differences” approach. Because some studies refer to differences between men and women as a “gender” difference, we also included this term (“*gender difference*^∗^”) in the category of sex studies. As an additional category, we included studies that more broadly considered *gender* issues capturing more subtle meanings that societies and individuals ascribe to male and female categories in the context of psychosocial risks. In this count we included studies looking at gender stereotypes and roles with the following terms as keywords: *gender OR masculine^∗^ OR feminine^∗^ OR* “*sex discrimination*” *OR* “*sex roles*.”

We contend that it is important to comprehensively distinguish between the components of sex and gender but we additionally acknowledge the theoretical difference between an inclusive gender perspective and a more specific approach dealing with gender identity traits, captured through the gender identity, gender role orientation, and androgyny literature (Bem, 1974; Spence and Helmreich, 1978; Spence and Buckner, 2000). To do so, and given the specific relevance of gender identities in gender research (e.g., Bem, 1974; Spence and Buckner, 2000; Bourne and Maxwell, 2010; Gartzia et al., 2012b), we included an additional count capturing studies that explicitly looked at gender identity dimensions through the following terms as keywords: “*gender identity^∗^*” *OR (gender AND communion) OR (gender AND expressiveness) OR (gender AND agency) OR (gender AND instrumentality) OR* “*gender role orientation*” *OR* “*gender ideology*” (Bem, 1974; Spence and Helmreich, 1978). To avoid overlap between the sex, gender and gender identity categories, we forced our broad gender count to exclude the terms implicitly included in the other categories (i.e., by including the following code in the gender category: *NOT* “*gender identity^∗^*” *NOT* “*gender difference^∗^*” *NOT* “*gender role orientation*” *NOT* “*gender ideology*”).

## Psychinfo Analyses: Results Classified by Type of Risk

### Workplace Stress

Although there are many available approaches to the study of psychosocial risks at work, work-related stress is a central dimension of study for occupational safety and health research (see Milczarek et al., 2009). Stress generally refers to the emotional and physiological reactions to stressors (Zastrow, 1984; Maslach et al., 1996) and is the most commonly studied psychosocial risk at work (see Figure 1). Research shows that prolonged stress is associated with a variety of mental health problems and emotional responses that can impair employees’ effectiveness (Caughey, 1996; Collings and Murray, 1996; Taylor-Brown et al., 1982; Zastrow, 1984). Although issues of occupational stress, health and well-being have been mostly addressed from the perspective of physical (cf. Cooper et al., 1994) and mental health (cf. Cartwright and Cooper, 1993; Anderson and Grunert, 1997), addressing the study of psychosocial risks from the perspective of gender and emotion is relevant because work experiences inherently alter individuals emotionally, subsequently influencing their psychological and organizational responses. Indeed, stress at work is often defined as a “negative psychological state with cognitive and emotional components, and on its effects on the health of both individual employees and their organizations” (Cox et al., 2000).

**FIGURE 2:**
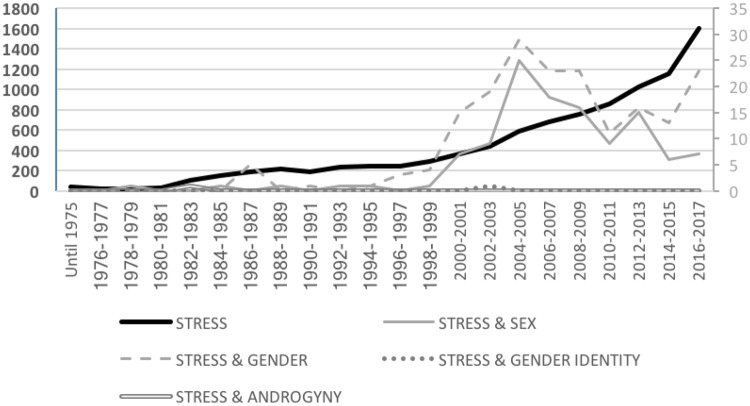
Total number of scientific articles per year about workplace stress by gender category. Given the substantially greater number of studies examining workplace stress than studies incorporating a gender perspective, data are presented in different scales. The axis on the left side of the figure represents the total number of articles for workplace stress (without gender-related keywords), whereas the axis on the right represents the total number of articles incorporating a gender perspective in any of our four gender categories (i.e., gender, sex differences, gender identity, or androgyny). The same rationale applies to Figures 3–6.

Following these literatures, the following terms were used for our category of workplace stress in our counting process of articles in the 1980–2017 period: *stress AND work OR burnout OR* “*work related stress*.” As shown in Figure 2, research in this field has increased gradually especially since the 2000s (growing from a total of 361 articles in 2000 to 1,599 in 2017). From a gender perspective, this pattern was also observed in relation to studies about workplace stress that considered gender generally and differences between men and women, although the number of studies considering these approaches are notably scarce (i.e., ranging from 15 articles in 2000 to 23 articles in 2017 in the gender category and 7 articles in 2000 to the same number in 2017 in the sex differences category). Remarkably, the interest in examining workplace stress from a gender perspective (i.e., including both general gender terms and a sex differences approach) showed a remarkable increase around 2005, probably due to the publication of a special issues or reviews about the topic. These counts point to the generally limited study of workplace stress from a gender perspective, being particularly absent the analysis of workplace stress from the perspective of gender identity traits (with a total of two studies in the overall 1980–2017 period).

These findings suggest that despite the growth of research in occupational health and the growing relevance given to the topic, gender research in general terms only represents a small proportion of articles within the workplace stress literature (1,97% of studies when considering gender broadly and 1,20% of studies when looking at sex differences), being particularly absent the consideration of how workplace stress can potentially be influenced by gender identities (0,02% of the total number of studies). As explained by social identity theories (Tajfel, 1978, 1982; Turner, 1987), social identities emerge from identifications with self-relevant groups and one’s social position in terms of gender (i.e., being male or female) constitutes one of the most influential dimensions in the development of gendered personality traits and identity (Spence and Helmreich, 1978; Bem, 1981; Tajfel and Turner, 1986). As such, gender identity traits (i.e., communion and agency) are central dimensions of the self with critical effects on behavior and emotional responses. Given the centrality of gendered traits for ones’ self-concept and behavior, the absence of studies looking at the connections of these gendered dimensions of personality with occupational stress is surprising.

The absence of a multidimensional gender approach in the workplace stress literature might help to explain previous inconsistencies in the field when considering the effects of gender variables indirectly (Hobfoll et al., 2003; Gyllensten and Palmer, 2005), suggesting that further research is needed (Christie and Schultz, 1998; Gianakos, 2002). Acknowledging the particular situation of discrimination that women experience at work (Kan et al., 2011; Hausmann et al., 2014), some studies have provided evidence that women’s experienced level of workplace stress is higher. A qualitative study investigating job stress among twelve managers in the English National Health Service reported that female managers were more at risk from managerial stressors compared to male managers (Jenkins and Palmer, 2004). In the same way, an Australian study showed that women experience higher levels of work stress than men, although they do not report worse mental health (Gardiner and Tiggemann, 1999). Michael et al. (2009) showed in a big sample of full-time working adults that women experience significantly higher levels of occupational stress. These findings are in line with previous research showing evidence of sex differences in both stressors and the severity of stress (Decker and Borgen, 1993). Contrasting this approach, other studies have shown similar levels of overall stress for male and female employees (e.g., Martocchio and O’Leary, 1989), underscoring the idea that factors like occupational level (i.e., being in a managerial position) and their connections to the subjective gendered experiences of men and women may play a stronger role (Spielberger and Reheiser, 1994). Likewise, it has been suggested that women experience higher levels of stress and burnout in general life but not specifically in the field of work (Etzion, 1984; Di Salvo et al., 1995).

In some cases, sex differences are argued to stem from women’s greater exposure to specific psychosocial stressors (Ritsner et al., 2001). Burnout is a particularly relevant form of work stress inherently liked to negative emotional responses including emotional exhaustion, depersonalization and reduced personal fulfillment (Maslach and Jackson, 1981). In particular, it refers to the emotional fatigue produced by the tasks of work, depersonalization to the distance treatment that is applied to people who have to attend and low personal fulfillment reflects the low professional self-esteem that accompanies the exercise of one’s profession (Maslach and Jackson, 1981). From a gender perspective, results are generally inconclusive about differences between men and women (Maslach et al., 2001). Going beyond a sex differences approach, Purvanova and Muros (2010) examined associations between gender and the two core components of work burnout (emotional exhaustion and depersonalization) and concluded that employees in gender-atypical occupations experience greater burnout and adverse health-related effects. However, women were more likely to report emotional exhaustion than men, whereas men were more likely to report depersonalization. Guthrie and Jones (2012) found similar results, with women and men experiencing similar differences in burnout levels, and differences emerging only by professional category.

### Workplace Abuse

Another important psychosocial risk captures abusive behaviors at work. According to Hackney and Perrewé (2018), workplace abuse is one of the most prevalent negative experiences of employees at work and can result in many dysfunctional outcomes for both employees and employers (Maslach et al., 1981; Leka et al., 2003). These dysfunctional consequences include attitudinal outcomes (e.g., satisfaction, commitment, and turnover intentions), behavioral outcomes (e.g., subsequent abuse or absenteeism), and well-being outcomes (e.g., psychological distress, job tension, burnout, and depression; for a review, see Hackney and Perrewé, 2018). Consistent with previous research (Bowling et al., 2015), workplace abuse can be considered an inclusive concept that encompasses different forms of physical and nonphysical mistreatment at the workplace by a variety of agents (e.g., supervisors, customers, and coworkers).

**FIGURE 3:**
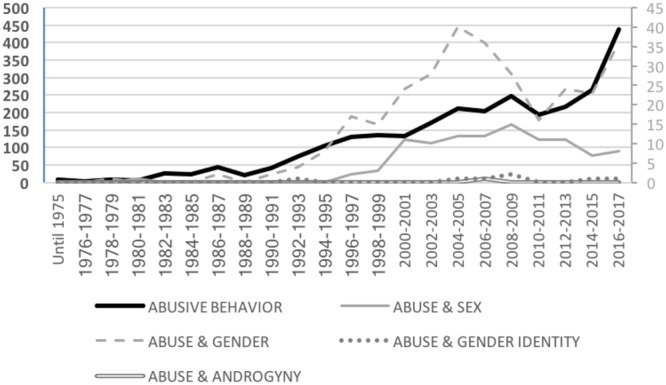
Total number of scientific articles per year about workplace abusive behavior by gender category.

Following this literature, in our count workplace abusive behavior includes a variety of related terms such as violence at work, workplace aggression, interpersonal mistreatment, mobbing, bullying, antisocial behavior, or workplace harassment (Hackney and Perrewé, 2018). In particular, the following terms were used in the counting process: “*workplace violence*” *OR* “*social undermining*” *OR* “*abusive supervision*” *OR* “*antisocial behavior in organization^∗^*” *OR* “*mobbing*” *OR* “*bullying at work*” OR “*sexual harassment*” *AND work*. As shown in Figure 3, research regarding abusive behaviors has grown in a regular pattern (i.e., ranging from 171 articles in 2000 to 437 articles in 2017), with a small decrease during the 2010–2015 period and a new rise afterward. In the same way, the number of articles taking gender issues into consideration has also increased since the 1990’s in relation to studies that have considered gender generally (i.e., increasing from 2 articles in 1990 to 36 articles in 2017) and to a lower extent in relation to studies that have focused on examining differences between men and women (increasing from 0 articles in 1990 to 8 articles in 2017). In both cases, increases in the number of studies were observed in the 2000’s, with subsequent decreases particularly in relation to the analyses of sex differences. As in relation to workplace stress, the number of studies examining workplace abuse from the perspective of gender identity traits is virtually absent.

Having only a limited number of studies explicitly examining the influence of gender dimensions in workplace abuse can be problematic because the scarce research looking at these connections has shown the potentially relevant effects of gender in how employees experience and produce harmful workplace experiences (see Sojo et al., 2016). For instance, given the noticeable prevalence of sexism in our society, women are more likely than men to be targets of sexual harassment and discrimination (Schmitt et al., 2002). The adverse impact of these behaviors is greater in male-dominated work contexts (O’Connell and Korabik, 2000) and when sexism is widely accepted as the norm (Settles et al., 2006). Note however that the inherently varied and multidimensional nature of hostile environments at work often makes it difficult to capture these effects. Whereas sexual harassment is often experienced by women, harmful workplace experiences come from many sources and take many different forms and so the prevalence of sex differences in relation to broader forms of violence at work is often less obvious. These entail structural factors of discrimination over women such as working conditions by which stereotyped forms of masculine behavior are reinforced (e.g., talking about football or good-looking girls; Collinson and Hearn, 1996; Powell and Graves, 2003; Rojo and Esteban, 2005) as well as organizational contexts in which sexual attributes of women are exhibited even when it is not related to the work that is being carried out (e.g., waitress or hostesses wearing skirts and sexually provocative clothing). Given the unconscious prevalence of many of these discriminatory norms and cultural ideals in organizations, sexual harassment against women often goes unnoticed and is socially legitimized (Gruber and Bjorn, 1982; Rospenda, 2002). In relation to this, the meta-analysis by Topa Cantisano et al. (2008) showed that organizational elements (in particular, job–gender context, social support, and organizational tolerance) had a critical role as antecedents that lead to sexual harassment. These harmful workplace experiences can add to the general pressures from general discrimination and demands associated with women’s role.

Mobbing is another specific form of violence, generally defined as “situations in the workplace where an employee persistently and over a long time perceives him- or her-self to be mistreated and abused by other organization members, and where the person in question finds it difficult to defend him/herself against these actions” (Nielsen and Einarsen, 2012). One aspect of great relevance from a gender perspective in the conceptualization of workplace mobbing and other forms of violence is the imbalance of power between the parties (Zapf and Einarsen, 2005), which might lead women to generally suffer from this psychosocial risk to a greater extent than men. Yet, meta-analytical data examining the relation between workplace bullying and mental health and examining the influence of demographic variables (e.g., including sex of the bullied person) have shown null effects of sex as a moderator in the abovementioned relation (Verkuil et al., 2015). Contrasting these findings, Tamres et al. (2002) reported in their meta-analysis when examining sex differences in coping behavior that women were more likely to use coping strategies that involve verbal expressions while men tend to avoid ventilating their problems with others in bullying situations. In relation to cyberbullying, the prevalence of sex differences has also been reported to be null (Cowan, 2018).

### Job Insecurity

**FIGURE 4:**
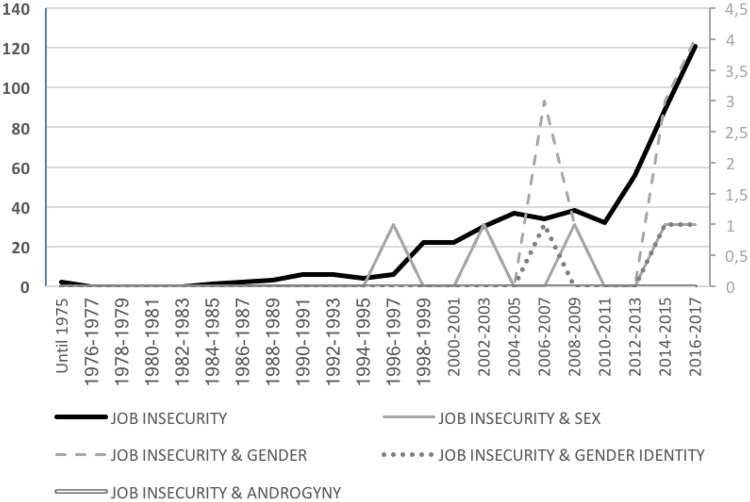
Total number of scientific articles per year about job insecurity by gender category.

Job insecurity, defined as a general concern about the continuance of work in the future or a perceived threat of several job characteristics such as one’s position or career opportunities (Cheng and Chan, 2008), is also a relevant source of work stress with inconclusive findings from a gender perspective. Because the need for security and stability is a fundamental human need (Greenhalgh and Rosenblatt, 1984; Leka et al., 2008), job insecurity has been described as one of the most important occupational risks. As our count of number of studies in the 1980–2017 period shows, however, job insecurity represents a relatively underesearched field of study, with only a total of 121 articles in 2017 (see Figure 1). Given the more focused study in this field, only the term “*job insecurity*” was used in the counting process for this category. It is interesting to note that research has also been generally limited from a gender perspective, with virtually no study explicitly incorporating gender-related variables as keywords in the 1980–2007 period (see Figure 4).

Contrasting these gaps in the job insecurity literature, and because job insecurity can derive from a wide variety of sources ranging from work conditions to leadership styles, the associations between job insecurity and gender should be further examined. Men usually have higher occupational mobility and promotions to managerial positions (Rosenblatt et al., 1999; Hausmann et al., 2014), so one view is that the threat of job loss should be less distressing to men than to women. Contrasting this view, there is evidence that men are more vulnerable to job insecurity than women because they are more sensitive to economic insecurity (Greenhalgh and Rosenblatt, 1984; De Witte, 1999) and the prevalence of the breadwinner role implicit in gender stereotypes. More recent meta-analytical evidence examining moderating effects of sex on the relationship between job insecurity and its consequences has shown null effects (Cheng and Chan, 2008). Among the explanations provided are female’s increasing commitment to their jobs and career roles (Bradley, 1997) and the greater similarities between the breadwinner roles and occupational mobility of men and women in today’s societies. Likewise, men have compared with women higher career-related expectations of workplace commitment in terms of paid work hours, which are subsequently more likely to be associated with well-being (Gartzia et al., 2012b).

Interestingly, previous research has acknowledged the specific relevance of gender identity traits in the subjective experience of job insecurity. A study by Gaunt and Benjamin (2007) showed that traditional men (in terms of gender role ideology) experience greater job insecurity than traditional women, whereas egalitarian men and women exhibit similar degrees of job insecurity. Interestingly, job insecurity in traditional men and in egalitarian men and women was also related to loss of control stress, financial stress and stress expressions at home, whereas traditional women suffered less job-related stress. These findings underscore the relevance of further examining within-sex differences and how gender ideologies and identities might influence the relationships between job-related factors and stress. Because job insecurity has been described as one of the most important occupational risks (Greenhalgh and Rosenblatt, 1984), examining these relations is critical.

### Emotional Labor

Emotional labor involves self-control of emotions by enhancing, faking, or suppressing emotions to modify the emotional expression, which can be detrimental for employees (Grandey, 2000; Miller et al., 2005). In today’s society and organizations employees are often required to appropriately respond to these social demands by for instance managing emotions and expressing organizationally desired emotions (Grandey, 2000). In particular, many organizations have established rules regarding the emotions that employees have to show to influence the emotions of clients and stakeholders (Moreno-Jiménez et al., 2010). Drawing from the literature in this field, the following terms were used in our counting process for the emotional labor category: “*emotional labor*” *OR* “*emotional work*.”

**FIGURE 5:**
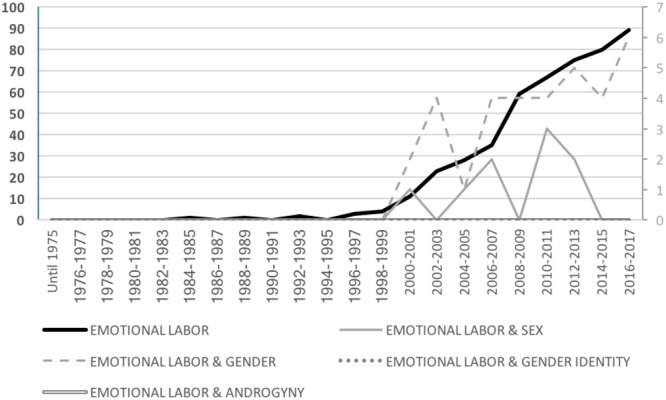
Total number of scientific articles per year about emotional labor by gender category.

As shown in Figure 5, no articles about emotional labor were developed until the 2000’s, when the term was fully introduced in the literature. Since then, research in this field has increased substantially (growing from a total of 11 articles in 2000 to 89 in 2017). From a gender perspective, this pattern is also observed in relation to studies about emotional labor that considered gender generally (i.e., increasing from 2 articles in 2000 to 6 articles in 2017). Although the explicit examination of sex differences in emotional labor is also present, findings show a substantially smaller interest in such approach with a particularly notorious decrease after 2010 (i.e., one article in 2000, 3 articles in 2010, and 0 articles in 2017). As in relation to the previous categories, the number of studies explicitly examining emotional labor from the perspective of gender identity traits is absent.

Although results are generally inconclusive in relation to sex differences in emotional labor (see Erickson and Ritter, 2001), there is some evidence that women are more likely to regulate emotions and suppress their true emotions in order to be effective at work, which has been linked to increased stress (Grandey, 2000). In general terms, women are socialized to behave in a warm and friendly manner (Deutsch, 1990) and are expected to express emotions (e.g., smile) to a greater extent than men in a variety of situations (e.g., Birnbaum et al., 1980; Deaux, 1985; LaFrance and Banaji, 1992). As such, women are expected to have a greater frequency of emotional display than men (Morris and Feldman, 1997). Interestingly, it has been argued that women may show many of these positive emotions because of the greater need for social approval implicit in their feminine gender role (Hoffman, 1972).

Acknowledging the differences between deep acting and surface acting, Johnson and Spector (2007) analyzed the relationship between deep acting and well-being related responses and found that sex was a moderator of the relationship between surface acting (“managing only observable expressions to obey display rules”) and the outcomes. For surface acting, women reported more detrimental outcomes than men (i.e., reduced affective well-being and job satisfaction, as well as greater emotional exhaustion). Yang and Guy (2014) found that expressing emotions that are not actually felt is not associated with job satisfaction or turnover for men, whereas it reduces such responses for women. Contrasting these effects, in a study about the effects of emotional labor for frontline service workers employed in the services sector, Wharton (1993) found that women in jobs requiring emotional labor were more satisfied than men in the same jobs, suggesting that women did not experience more negative consequences but even experienced psychological reward. Based on the results of our counting process of articles and to our knowledge, previous studies have not explicitly considered emotional labor from the perspective of gender identities. Likewise, the potential effects of emotional intelligence competences on how emotional labor influences work outcomes and employees’ well-being is unclear (Johnson and Spector, 2007), so additional research would be needed to better understand the connections between gender roles and identities, emotional competences and well-being outcomes resulting from emotional work.

### Work-Family Conflict

**FIGURE 6:**
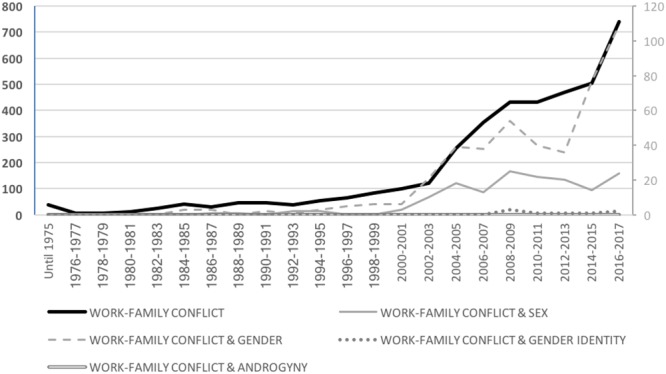
Total number of scientific articles per year about the work–family conflict by gender category.

Greenhaus and Beutell (1985) defined this particular type of conflict as “a form of interrole conflict in which the role pressures from the work and family domains are mutually incompatible in some respect” (p. 77). In this approach, the work-family conflict is both bi-directional and multi-dimensional, such that one’s family life can interfere with work, and vice versa. Following the rich literature in this field, the following terms were used in the counting process: “*work–family*” *OR* “*work-life*” *OR* “*family friendly*.” As shown in Figure 6, research in this field increased gradually especially since the 2000s, when the concepts were more consistently established in the organizational behavior literature (growing from a total of 99 articles in 2000 to 740 in 2017). From a gender perspective, this pattern is also observed in relation to studies about workplace stress that have considered gender generally (i.e., increasing from 6 articles in 2000 to 109 articles in 2017) and to a substantially lower extent in relation to studies that have focused on examining differences between men and women (increasing from 3 articles in 2000 to 24 articles in 2017). In contrast, the number of studies examining workplace stress from the perspective of gender identity traits is virtually absent.

Although gender has been a variable of interest in numerous work–family studies (Eby et al., 2005; Bianchi and Milkie, 2010), the specific way in which gender is related to work-life balance is unclear and sex differences are inconsistent, as revealed in several reviews of the work–family literature (e.g., Frone, 2003; Eby et al., 2005; Korabik et al., 2008). Because there is accumulated evidence that this form of role conflict influences emotional responses at work such as emotional exhaustion, due to the negative effects derived from the tension produced by the incompatible pressures from the work and family domains (e.g., Lingard and Francis, 2005; Karatepe and Sokmen, 2006; Glaser and Hecht, 2013), understanding these relations is critical. A meta-analysis by Shockley et al. (2017) showed that men and women showed similar work–family experiences overall, regardless of the specific subgroups that were examined in relation to characteristics such as the nature of the job, working time (full time vs. part-time), parenthood (parents vs. not parents), and the incomes of the partner (dual-earner couple vs. single-earner couple). To avoid a simplistic interpretation of these findings, Shockley et al. (2017) underscored the many complexities and intricacies involved in this phenomenon that required further study, as for instance looking at moderators like job autonomy and family boundaries. Because these spheres interrelate comprising both work-to-family and family-to-work demands (Greenhaus and Beutell, 1985), the specific ways in which these two associations are influenced by gender stereotypes and identities might also vary.

## Emotional Androgyny: A Potential Resource to Reduce Psychosocial Risks at Work?

Our count of scientific articles about the most commonly studied psychosocial risks at work in the in the 1980–2017 period (namely workplace stress, abusive behavior, job insecurity, emotional labor, and the work–family conflict) revealed a notorious absence of research explicitly examining the connections between gender and psychosocial risks at work, and particularly in relation to communal and agentic gendered traits. Likewise, our review of the literature examining the gendered nature of emotional competences revealed remaining challenges in our understanding of how gendered functions and identities interrelate to influence emotional responses and processes at work. Because emotional competences and regulation strategies have emerged as a key resource to reduce psychosocial risks in a variety of studies (Grandey and Brauburger, 2002; Pugh, 2002; Coté, 2005), further research capturing these complexities from a gender perspective is likely to be useful. In particular, given the numerous inconsistencies in the literature examining sex differences in psychosocial risks, we suggest that these associations can be better understood from a comprehensive gender perspective that goes beyond sex differences and acknowledges the many complexities involved in gendered variables and prescriptions.

Emotional intelligence researchers’ holistic perspective of emotional competences and gender identity researchers’ integrative approach of identity traits (see Mayer et al., 2008; Wood and Eagly, 2015, respectively, for comprehensive reviews) provide closely related viewpoints in relation to how individuals can successfully regulate emotions and behavior. Both research areas attempt to explain how integrative approaches of human behavior that incorporate a diverse range of socioemotional dimensions influence work experiences, although they differ in their focus. Emotion regulation and emotional intelligence research is concerned with how emotional life influences subjective experiences at work and how a wider range of emotional competences and regulation strategies leads to more adaptive behavior (e.g., Gross, 1999; Bar-On et al., 2000; Coté, 2005; Mayer et al., 2008). Gender identity research in turn explains how integrating counter-stereotypical elements into one’s identity can help people develop healthier emotional responses. Adding to these literatures, the specific issue of how gender might help understand psychosocial risks at work has too often been oversimplified, so a perspective that acknowledges both self-oriented (i.e., agentic or stereotypically masculine) and other-oriented (i.e., communal or stereotypically feminine) emotional competences can be critical to understand psychosocial risks at work.

The concept of androgyny and its relation to life adjustment has been often criticized based on methodological concerns about interactions of agency and communion as measured with the Bem Sex Role Inventory (BSRI, Bem, 1974) and the Personal Attributes Questionnaire (PAQ, Spence and Helmreich, 1978) and the idea that societies are inherently gendered and prescribe different roles and traits for men and women, making it difficult to develop androgyny in the practice (e.g., Whitley, 1983; Egan and Perry, 2001). Derived from these concerns, many researchers abandoned the field because of a seemingly lack of agreement about its operationalization and applicability. Nonetheless, the concept of androgyny provides theoretical basis to understand gender behavioral flexibility and how having a wider range of responses serve to adapt to the environment. Furthermore, the gender literature contains indications that psychological androgyny is associated with mental and social well-being.

The early literature on sex role orientations (i.e., gender identity) showed that psychological androgyny is related to higher self-esteem, relationship satisfaction, better physical health, and lower loneliness (see, e.g., Bem, 1974; Ickes, 1993; Helgeson, 1994). In contrast, there is evidence that people with stereotyped identities display poorer psychological adjustment and wellbeing (Osofsky and Osofsky, 1972; Whitley, 1983; Williams and D’Alessandro, 1994). Recent studies have also shown that combinations of communion and are relevant predictors of behavior beyond sex in dimensions generally associated with subjective well-being. In the particular domain of emotion, employees with counter-stereotypical gender profiles (i.e., androgynous employees) have shown to be better able than other individuals with stereotyped identities to understand and regulate emotions as measured with both self-report and ability-based measures (Gartzia and van Engen, 2012; Gartzia et al., 2012a). Likewise, gender identity traits help to explain sex differences in a wide range of behaviors and emotional dimensions including verbal processing or processing tasks of facial emotions (Bourne and Maxwell, 2010). The incremental validity of gender traits over sex predicting psychological and emotional responses has also been demonstrated using neurological correlates (Weekes et al., 1995; Bourne and Gray, 2009).

Previous research has also addressed the issue of the possible advantages of androgyny for organizations. For instance, there is accumulated evidence in the organizational behavior literature that groups with members who are able to fill both task oriented and people-oriented roles (i.e., instrumental and expressive) are more cohesive and perform more effectively (Mudrack and Farrell, 1995). Indeed, as Parsons and Bales pointed from their functional perspective of team roles (1953), there is usually an “equilibrium problem” of establishing cyclic patterns of interaction that move the group forward to accomplish the task, and patterns of interaction that restore the internal socioemotional balance disturbed by the pursuit of the task. From this perspective, task and relationship roles are a natural consequence of these two partly conflicting demands and implies that a combination of both communal and agentic traits are required for effective functioning (Bales, 1953; Belbin, 1993). Drawing from this research, recent studies have shown that androgynous individuals are more effective in several organizational functions (e.g., Gartzia and van Engen, 2012; Gartzia et al., 2012a). For instance, Gartzia and Van Knippenberg (2015) showed that compared to male leaders with stereotypically masculine agentic profiles, male leaders with communal traits (i.e., androgynous) increased behavioral cooperation of team members participating in a prisoner’s dilemma game.

In a study with Israeli employees rating their managers in relation to gender role orientation (perceived communion and agency) and leadership styles as measured with the MLQ, Kark et al. (2012) showed that androgyny was more strongly related to transformational leadership and followers’ identification than other personality dimensions, and furthermore that gender traits showed incremental validity over sex predicting such leadership styles. Relatedly, Gartzia and van Engen (2012), showed that sex differences favoring women in individualized consideration, positive contingent reward and emotional intelligence were at least in part explained by male leaders’ lower identification with communal traits. These findings are consistent with Zaccaro’s (2007) suggestion that the ability of leaders to display a mix of different traits is critical for effective leadership as it allows having an expansive behavioral repertoire and adapting one’s behavior as the situation changes. This standpoint ultimately provides relevant counterpoints to the oft-quoted *think manager-think male* perspective in leadership (Koenig et al., 2011).

Drawing from this literature, we propose emotional androgyny as a promising dimension in understanding and reducing psychosocial risks at work. Emotional androgyny can be understood here as achieving a balance between stereotypically masculine and stereotypically feminine traits related to a broad variety of perceptual, affective, and behavioral emotional dimensions that can potentially reduce the effects of psychosocial hazards at work. Note that we do not attempt an exhaustive examination of the androgyny concept nor do we attempt to address all the associations that may exist between gender and emotional competences, since this is addressed in several other publications and debates (e.g., see Brody and Hall, 2000; Fischer and Manstead, 2000; Bindu and Thomas, 2006; Gartzia and van Engen, 2012; Gartzia et al., 2012a). We rather focus on providing an updated reflection of the theoretical and empirical relevance of the topic and its potential applicability to dealing with the many challenges associated with preventing psychosocial risks at work. This process is twofold.

At the organizational/structural level, developing more flexible and “androgynous” viewpoints may prove useful to reduce job strain by for instance promoting leadership styles that combine agentic traits and emotional competences (e.g., assertiveness, self-confidence, and regulation of negative emotions such as guilt) with communal traits and emotional competences (e.g., empathy, listening skills and attention to, understanding and regulation of others’ emotions). Because leaders’ traits and emotion regulation strategies are critical for employees’ psychological responses and well-being at work (see Mayer et al., 2008; Hackney and Perrewé, 2018) as well as for their subsequent development of emotion regulation strategies in the organization (Mawritz et al., 2017), leaders’ androgynous profiles might help reduce employees’ psychosocial hazards and improve their physical, mental, and social well-being. Because leaders are generally responsible for applying organizational norms and procedures, their action and attitudes are detrimental to the application of such procedures (Folger and Bies, 1989; Foreman and Whetten, 2002) and thus leaders’ capacity to affect the physical, mental and social well-being of workers is potentially linked to a wider number of occupational risks (e.g., work-life balance policies and culture, strategic decisions about job conditions, or regulations about exemplary behavior at work). The incorporation of more androgynous managerial profiles might be particularly useful in dynamic and complex work environments in which competition and innovation is critical. Because these dynamic organizational environments often lead to failure experiences and negative emotions that worsen employees’ motivation and learning (Shepherd et al., 2011), efforts to successfully combine communal and agentic qualities can become critical.

Second, at the individual level, developing more flexible and “androgynous” identities will assist health concerns such as work-related stress and its consequences by providing employees with a richer umbrella of emotional competences that help them cope with the demands placed on them by their work. As we have argued, individuals with stereotyped identities have limited emotional competences because stereotypically feminine (i.e., communal) and stereotypically masculine (i.e., agentic) traits are associated with different emotional competences – communion is related to emotional attention and regulation of emotion in others whereas agency is related to one’s emotional repair (Gartzia et al., 2012b). The occupational health literature has shown that employees’ emotional resources and regulation strategies are critical to cope with organizational demands and psychosocial risks (Ashforth and Humphrey, 1993; Grandey and Brauburger, 2002; Pugh, 2002; Coté, 2005; Hackney and Perrewé, 2018) and so acknowledging the gendered nature of these strategies and their subsequent influence on the experience, expression, and regulation of emotions opens the door to new questions and insight about how to overcome current challenges in how men and women differently experience occupational risks.

## Concluding Remarks

The aim of the current paper was to provide a critical approach about the multidimensional ways in which gender and psychosocial risks interrelate, focusing on emotional competences and their dynamic gendered nature as a useful framework to address the many challenges that these associations pose. With the relevance of emotional competences to prevent and deal with psychosocial risks and work-related stress (Ashforth and Humphrey, 1993; Grandey and Brauburger, 2002; Pugh, 2002; Coté, 2005), developing a critical approach to understand the multifaceted associations between emotion, organizational demands, and gender is of potential importance for the work and stress literature. However, our analysis of scientific articles in psychosocial risks revealed that analyses about the multidimensional ways in which gender is associated with employees’ hazards at work have been clearly narrow. We gave specific attention to gendered dimensions that are open to change, focusing on the notion of gender identities and androgyny. Androgynous individuals may have the potential to develop the wide range of emotional competences that are required to deal with and improve emotional experiences at work. Thus, the integration of this approach allows us to pose new theoretical and methodological directions to further understand the psychosocial processes that affect women’s and men’s stressful workplace experiences. We hope that these propositions will foster the development of cumulative knowledge of the gendered nature of occupational risks at work in a way that a sex differences approach alone might not do.

## Author Contributions

LG conceived the original idea and the computational framework. JB encouraged LG to perform counts about the scientific literature and supervised the findings of this work. JP developed the analyses and performed literature reviews. LG took the lead in writing the manuscript. All authors discussed the results and contributed to the final manuscript.

## Conflict of Interest Statement

The authors declare that the research was conducted in the absence of any commercial or financial relationships that could be construed as a potential conflict of interest.

## References

[B1] Acker G. M. (1999). The impact of clients’ mental illness on social workers’ job satisfaction and burnout. *Health Soc. Work* 24 112–119. 10.1093/hsw/24.2.112 10340161

[B2] Allen J. G. Haccoun D. M. (1976). Sex differences in emotionality: a multidimensional approach. *Hum. Relat.* 29 711–722. 10.1177/001872677602900801

[B3] Anderson R. C. Grunert B. K. (1997). A cognitive behavioral approach to the treatment of post-traumatic stress disorder after work-related trauma. *Prof. Saf.* 42:39.

[B4] Ashforth B. E. Humphrey R. H. (1993). Emotional labor in service roles: the influence of identity. *Acad. Manage. Rev.* 18 88–115. 10.3109/09638237.2012.734656 23216225

[B5] Avolio B. J. Gardner W. L. (2005). Authentic leadership development: getting to the root of positive forms of leadership. *Leadersh. Q.* 16 315–338. 10.1016/j.leaqua.2005.03.001

[B6] Babin B. J. Boles J. S. (1996). The effects of perceived co-worker involvement and supervisor support on service provider role stress, performance and job satisfaction. *J. Retailing* 72 57–75. 10.1016/S0022-4359(96)90005-6

[B7] Bales R. F. (1953). “The equilibrium problem in small groups,” in *Working Papers in the Theory of Action*, eds Parsons T. Bales R. F. Shils E. A. (Westport, CT: Greenwood Press).

[B8] Bandura A. (1983). Psychological mechanisms of aggression. *Aggress. Theor. Empir. Rev.* 1 1–40.

[B9] Bar-On R. Brown J. M. Kirkcaldy B. D. Thome E. P. (2000). Emotional expression and implications for occupational stress; an application of the Emotional Quotient Inventory (EQ-i). *Personal. Individ. Differ.* 28 1107–1118. 10.1016/S0191-8869(99)00160-9

[B10] Baron-Cohen S. (2002). The extreme male brain theory of autism. *Trends Cogn. Sci.* 6 248–254. 10.1016/S1364-6613(02)01904-6 12039606

[B11] Baron-Cohen S. (2003). *The Essential Difference: Men, Women and the Extreme Male Brain.* London: Allen Lane

[B12] Baumeister R. F. (2002). Yielding to temptation: self-control failure, impulsive purchasing, and consumer behavior. *J. Consum. Res.* 28 670–676. 10.1086/338209

[B13] Belbin R. M. (1993). A reply to the belbin team-role self-perception inventory by furnham, steele and pendleton. *J. Occup. Organ. Psychol.* 66 259–260. 10.1111/j.2044-8325.1993.tb00536.x

[B14] Bem S. L. (1974). The measurement of psychological androgyny. *J. Consult. Clin. Psychol.* 42:155 10.1037/h0036215 4823550

[B15] Bem S. L. (1981). Gender schema theory: a cognitive account of sex typing. *Psychol. Rev.* 88 354–364. 10.1037/0033-295X.88.4.354

[B16] Bianchi S. M. Milkie M. A. (2010). Work and family research in the first decade of the 21st century. *J. Marriage Fam.* 72 705–725. 10.1111/j.1741-3737.2010.00726.x

[B17] Bindu P. Thomas I. (2006). Gender differences in emotional intelligence. *Psychol. Stud. Univ. Calicut* 51:261.

[B18] Birnbaum D. W. Nosanchuk T. A. Croll W. L. (1980). Children’s stereotypes about sex differences in emotionality. *Sex Roles* 6 435–443. 10.1007/BF00287363

[B19] Bittman M. Hill T. Thomson C. (2007). The impact of caring on informal carers’ employment, income and earnings: a longitudinal approach. *Aust. J. Soc. Issues* 42 255–272. 10.1002/j.1839-4655.2007.tb00053.x

[B20] Blanchard K. H. (1993). The blanchard management report. *Management* 4:1.

[B21] Bond S. A. Tuckey M. R. Dollard M. F. (2010). Psychosocial safety climate, workplace bullying, and symptoms of posttraumatic stress. *Organ. Dev. J.* 28 37–56.

[B22] Bono J. E. Ilies R. (2006). Charisma, positive emotions and mood contagion. *Leadersh. Q.* 17 317–334. 10.1016/j.leaqua.2006.04.008

[B23] Borland J. J. (1981). Burnout among workers and administrators. *Health Soc. Work* 6 73–78. 10.1093/hsw/6.1.73 7215992

[B24] Bourne V. J. Gray D. L. (2009). Hormone exposure and functional lateralisation: examining the contributions of prenatal and later life hormonal exposure. *Psychoneuroendocrinology* 34 1214–1221. 10.1016/j.psyneuen.2009.03.010 19375867

[B25] Bourne V. J. Maxwell A. M. (2010). Examining the sex difference in lateralisation for processing facial emotion: does biological sex or psychological gender identity matter? *Neuropsychologia* 48 1289–1294. 10.1016/j.neuropsychologia.2009.12.032 20036677

[B26] Bowling N. A. Camus K. A. Blackmore C. E. (2015). “Conceptualizing and measuring workplace abuse: implications for the study of abuse’s predictors and consequences,” in *Mistreatment in Organizations*, eds Perrewe P L. Halbesleben J R. B. Rosen C C. (Bingley: Emerald Group Publishing Limited), 225–263 10.1108/S1479-355520150000013008

[B27] Bradley H. (1997). “Gender and change in employment: feminization and its effects,” in *The Changing Shape of Work*, ed. Brown R. (London: Macmillan).

[B28] Brief A. P. Weiss H. M. (2002). Organizational behavior: affect in the workplace. *Annu. Rev. Psychol.* 53 279–307. 10.1146/annurev.psych.53.100901.135156 11752487

[B29] Briner R. B. (1994). Stress and well-being at work-assessments and interventions for occupational mental-health. *J. Occup. Organ. Psychol.* 67 183–184.

[B30] Brody L. Hall J.A. (2000), “Gender, emotion, and expression,” in *Handbook of Emotions*, eds Lewis M. Jeannette H. J. (New York, NY: Guilford Press), 338–349.

[B31] Brody L. R. Hall J. A. (2008). “Gender and emotion in context,” in *Handbook of Emotions*, eds Lewis M. Jeannette H. J. (New York, NY: Guilford Press), 395–405.

[B32] Brody L. R. (1997). Gender and emotion: beyond stereotypes. *J. Soc. Issues* 53 369–393. 10.1111/j.1540-4560.1997.tb02448.x

[B33] Cartwright S. Cooper C. L. (1993). The role of culture compatibility in successful organizational marriage. *Acad. Manag. Perspect.* 7 57–70. 10.5465/ame.1993.9411302324

[B34] Caughey J. (1996). Psychological distress in staff of a social services district office: a pilot study. *Br. J. Soc. Work* 26 389–398. 10.1093/oxfordjournals.bjsw.a011102

[B35] Cheng G. H. L. Chan D. K. S. (2008). Who suffers more from job insecurity? A meta analytic review. *Applied Psychology*, 57 272–303. 10.1111/j.1464-0597.2007.00312.x

[B36] Chirico F. (2017). The forgotten realm of the new and emerging psychosocial risk factors. *J. Occup. Health* 59 433–435. 10.1539/joh.17-0111-OP 28819083 PMC5635152

[B37] Christie W. G. Schultz P. H. (1998). *Dealer Markets Under Stress: The Performance of Nasdaq Market Makers During the November 15, 1991, Market Break. In Stock Market Policy Since the 1987 Crash*, Boston, MA: Springer, 23–47. 10.1007/978-1-4615-5707-4_3

[B38] Ciarrochi J. V. Chan A. Y. Caputi P. (2000). A critical evaluation of the emotional intelligence construct. *Personal. Individ. Differ.* 28 539–561. 10.1016/S0191-8869(99)00119-1

[B39] Cifre E. Vera M. Rodríguez-Sánchez A. M. Pastor M. C. (2013). Job-person fit and well-being from a gender perspective. *Rev. Psicol. Trabajo Organ.* 29 161–168. 10.5093/tr2013a22

[B40] Cifre E. Vera M. Signani F. (2015). Mujeres y hombres en el trabajo: analizando el estrés ocupacional y el bienestar desde una perspectiva de género. *Rev. Puertorriqueña Psicol.* 26 1–20.

[B41] Coleman B. C. (1997). Job-related injuries, illness take heavy toll. *Houston Chronicle*, 28th July.

[B42] Collings J. A. Murray P. J. (1996). Predictors of stress amongst social workers: an empirical study. *Br. J. Soc. Work* 26 375–387. 10.1093/oxfordjournals.bjsw.a011101

[B43] Collinson D. Hearn J. (eds) (1996). *Men as Managers, Managers as Men: Critical Perspectives on Men, Masculinities and Managements.* London: Sage 10.4135/9781446280102

[B44] Conway M. M. (2001). Women and political participation. *Polit. Sci. Polit.* 34 231–233. 10.1017/S1049096501000385

[B45] Cooper C. L. Cartwright S. (1994). Healthy mind; healthy organization—a proactive approach to occupational stress. *Hum. Relat.* 47 455–471. 10.1177/001872679404700405

[B46] Cooper C. L. Kirkaldy B. D. Brown J. (1994). A model of job stress and physical health: the role of individual differences. *Personal. Individ. Differ.* 16 653–655. 10.1080/10705500701783934 18444020

[B47] Cornelius R. R. Averill J. R. (1980). The influence of various types of control on psychophysiological stress reactions. *J. Res. Personal.* 14 503–517. 10.1016/0092-6566(80)90008-2 12669503

[B48] Coté S. (2005). A social interaction model of the effects of emotion regulation on work strain. *Acad. Manag. Rev.* 30 509–530. 10.5271/sjweh.3557 26960179

[B49] Cowan R. L. (2018). “When Workplace Bullying and Mobbing Occur: The Impact on Organizations,”in *Workplace Bullying and Mobbing in the United States 2 Volume* eds Duffy M. Yamada D. C. (Santa Barbara, CA: ABC-CLIO LLC)

[B50] Cox T. Griffiths A. Rial-González E. (2000). *Research on Work-Related Stress.* Luxembourg: European Communities.

[B51] De Witte H. D. (1999). Job insecurity and psychological well-being: review of the literature and exploration of some unresolved issues. *Eur. J. Work Organ. Psychol.* 8 155–177. 10.1080/135943299398302

[B52] Deaux K. (1985). Sex and gender. *Annu. Rev. Psychol.* 36 49–81. 10.1146/annurev.ps.36.020185.000405

[B53] Decker P. J. Borgen F. H. (1993). Dimensions of work appraisal: stress, strain, coping, job satisfaction, and negative affectivity. *J. Couns. Psychol.* 40 470–478. 10.1037/0022-0167.40.4.470

[B54] Deutsch M. (1990). Psychological roots of moral exclusion. *J. Soc. Issues* 46 21–25. 10.1111/j.1540-4560.1990.tb00269.x

[B55] Di Salvo V. Lubbers C. Rossi A. M. Lewis J. (1995). “Unstructured perceptions of work-related stress: An exploratory qualitative study,” in *Series in Health Psychology and Behavioral Medicine. Occupational Stress: A Handbook*, eds Crandall R. Perrewé P. L. (Philadelphia, PA: Taylor and Francis), 39–50.

[B56] Eagly A. H. Eaton A. Rose S. M. Riger S. McHugh M. C. (2012). Feminism and psychology: analysis of a half-century of research on women and gender. *Am. Psychol.* 67 211–230. 10.1037/a0027260 22369245

[B57] Eagly A. H. Gartzia L. Carli L. L. (2014). *Female Advantage: Revisited. In The Oxford Handbook of Gender in Organizations.* Oxford: Oxford University Press.

[B58] Eby L. T. Casper W. J. Lockwood A. Bordeaux C. Brinley A. (2005). Work and family research in IO/OB: content analysis and review of the literature (1980–2002). *J. Vocat. Behav.* 66 124–197. 10.1016/j.jvb.2003.11.003

[B59] Egan M. (1993). Resilience at the front lines: hospital social work with AIDS patients and burnout. *Soc. Work Health Care* 18 109–125. 10.1300/J010v18n02_07 8332938

[B60] Egan S. K. Perry D. G. (2001). Gender identity: a multidimensional analysis with implications for psychosocial adjustment. *Dev. Psychol.* 37 451–463. 10.1037/0012-1649.37.4.451 11444482

[B61] Erickson R. J. Ritter C. (2001). Emotional labor, burnout, and inauthenticity: does gender matter? *Soc. Psychol. Q.* 64 146–163. 10.2307/3090130

[B62] Etzion D. (1984). Moderating effect of social support on the stress–burnout relationship. *J. Appl. Psychol.* 69 615–622. 10.1037/0021-9010.69.4.615 6511711

[B63] European Commission (2015). *Visions for Gender Equality.* Luxembourg: Publications Office of the European Union 10.2838/00811

[B64] European Foundation for the Improvement of Living and Working Conditions (2007). *Work-Related Stress.* Loughlinstown: European Foundation for the Improvement of Living and Working Conditions

[B65] Fineman M. A. (2010). “The vulnerable subject: anchoring equality in the human condition” in *Transcending the Boundaries of Law*, ed. Fineman M. A. (London: Routledge-Cavendish), 177–191.

[B66] Fischer A. H. Manstead A. S. (2000). The relation between gender and emotions in different cultures. *Gend. Emot. Soc. Psychol. Perspect.* 1 71–94. 10.1017/CBO9780511628191.005

[B67] Folger R. Bies R. J. (1989). Managerial responsibilities and procedural justice. *Empl. Responsib. Rights J.* 2 79–90. 10.1007/BF01384939

[B68] Foreman P. Whetten D. A. (2002). Members’ identification with multiple-identity organizations. *Organ. Sci.* 13 618–635. 10.1037/apl0000344 30091620

[B69] Frijda N. H. Mesquita B. (1994). “The social roles and functions of emotions,” in *Emotion and Culture: Empirical Studies of Mutual Influence*, eds Kitayama S. Markus H. R. (Washington, DC: American Psychological Association), 51–87. 10.1037/10152-002

[B70] Frijda N. H. (1986). *The Emotions.* Cambridge: Cambridge University Press.

[B71] Frone M. R. (2003). “Work-family balance,” in *Handbook of Occupational Health Psychology*, eds Quick J. C. Tetrick L. E. (Washington, DC: American Psychological Association), 143–162 10.1037/10474-007

[B72] Ganster D. C. Schaubroeck J. (1991). Work stress and employee health. *J. Manag.* 17 235–271. 10.1177/014920639101700202

[B73] Gardiner M. Tiggemann M. (1999). Gender differences in leadership style, job stress and mental health in male and female dominated industries. *J. Occup. Organ. Psychol.* 72 301–315. 10.1348/096317999166699

[B74] Gartzia L. Aritzeta A. Balluerka N. Barberá E. (2012a). Inteligencia emocional y género: más allá de las diferencias sexuales. *Anal. Psicol.* 28 567–575

[B75] Gartzia L. Ryan M. K. Balluerka N. Aritzeta A. (2012b). Think crisis–think female: further evidence. *Eur. J. Work Organ. Psychol.* 21 603–628. 10.1097/TA.0000000000001405 28240673

[B76] Gartzia L. López-Zafra E. (2014). Gender research in spanish psychology: an overview for international readers. *Sex Roles* 70 445–456. 10.1007/s11199-014-0380-x

[B77] Gartzia L. Lopez-Zafra E. (2016). Gender research in spanish psychology, part ii: progress and complexities in the european context. *Sex Roles* 74 97–106. 10.1007/s11199-015-0567-9

[B78] Gartzia L. van Engen M. (2012). Are (male) leaders “feminine” enough? gendered traits of identity as mediators of sex differences in leadership styles. *Gend. Manag. Int. J.* 27 296–314. 10.1108/17542411211252624

[B79] Gartzia L. Van Knippenberg D. (2015). Too masculine, too bad: effects of communion on leaders’ promotion of cooperation. *Group Organ. Manag.* 41 458–490. 10.1177/1059601115583580

[B80] Gaunt R. Benjamin O. (2007). Job insecurity, stress and gender: the moderating role of gender ideology. *Community Work Fam.* 10 341–355. 10.1080/13668800701456336

[B81] Gianakos I. (2002). Predictors of coping with work stress: The influences of sex, gender role, social desirability, and locus of control. *Sex Roles* 46 149–158. 10.1023/A:1019675218338

[B82] Glaser W. Hecht T. D. (2013). Work-family conflicts, threat-appraisal, self-efficacy and emotional exhaustion. *J. Manage. Psychol.* 28 164–182. 10.1108/02683941311300685

[B83] Grandey A. A. (2000). Emotional regulation in the workplace: a new way to conceptualize emotional labor. *J. Occup. Health Psychol.* 5:95. 10.1037/1076-8998.5.1.95 10658889

[B84] Grandey A. A. Brauburger A. L. (2002). The emotion regulation behind the customer service smile. *Emot. Workplace Unders. Struct. Role Emot. Organ. Behav.* 260:294.

[B85] Greenhalgh L. Rosenblatt Z. (1984). Job insecurity: toward conceptual clarity. *Acad. Manage. Rev.* 9 438–448. 10.5465/amr.1984.4279673

[B86] Greenhaus J. Beutell N. (1985). Sources of conflict between work and family roles. *Acad. Manage. Rev.*,10 76–88. 10.5465/amr.1985.4277352

[B87] Gross J. J. (1999). Emotion regulation: past, present, future. *Cogn. Emot.* 13 551–573. 10.1080/026999399379186

[B88] Gruber J. E. Bjorn L. (1982). Blue-collar blues: The sexual harassment of women autoworkers. *Work Occup.* 9 271–298. 10.1177/0730888482009003002

[B89] Guastello D. D. Guastello S. J. (2003). Androgyny, gender role behavior, and emotional intelligence among college students and their parents. *Sex Roles* 49 663–673. 10.1023/B:SERS.0000003136.67714.04

[B90] Guthrie C. P. Jones A. III. (2012). Job burnout in public accounting: understanding gender differences. *J. Manag. Issues* 24 390–411.

[B91] Gyllensten K. Palmer S. (2005). Can coaching reduce workplace stress. *Coach. Psychol.* 1 15–17.

[B92] Hackney K. J. Perrewé P. L. (2018). A review of abusive behaviors at work: the development of a process model for studying abuse. *Organ. Psychol. Rev.* 8 70–92. 10.1177/2041386618755724

[B93] Hall J. A. Rosenthal R. Archer D. DiMatteo M. R. Rogers P. L. (1978). Decoding wordless messages. *Hum. Nat.* 1 68–75.

[B94] Hausmann R. Hidalgo C. A. Bustos S. Coscia M. Simoes A. Yildirim M. A. (2014). *The Atlas of Economic Complexity: Mapping Paths to Prosperity.* Cambridge, MA: Mit Press.

[B95] Helgeson V. S. (1994). Relation of agency and communion to well-being: evidence and potential explanations. *Psychol. Bull.* 116 412–428. 10.1037/0033-2909.116.3.412

[B96] Hobfoll S. E. Johnson R. J. Ennis N. Jackson A. P. (2003). Resource loss, resource gain, and emotional outcomes among inner city women. *J. Pers. Soc. Psychol.* 84 632–643. 10.1037/0022-3514.84.3.632 12635922

[B97] Hoffman L. W. (1972). Early childhood experiences and women’s achievement motives. *J. Soc. Issues* 28 129–155. 10.1111/j.1540-4560.1972.tb00022.x

[B98] Hoke G. E. J. (1997). Ergonomics: one size does not fit all. *Telemarketing* 16 28–31.

[B99] Hopkins M. M. Bilimoria D. (2008). Social and emotional competencies predicting success for male and female executives. *J. Manag. Dev.* 27 13–35. 10.1108/02621710810840749

[B100] Hornstein H. A. (1996). *Brutal Bosses and their Prey.* New York, NY: Riverhead books.

[B101] Hyde J. S. (2005). The gender similarities hypothesis. *Am. Psychol.* 60 581–592. 10.1037/0003-066X.60.6.581 16173891

[B102] Ickes W. (1993). Empathic accuracy. *J. Pers.* 61 587–610. 10.1111/j.1467-6494.1993.tb00783.x

[B103] Ickes W. Gesn P. R. Graham T. (2000). Gender differences in empathic accuracy: differential ability or differential motivation?. *Pers. Relat.* 7 95–109. 10.1111/j.1475-6811.2000.tb00006.x

[B104] Jenkins D. Palmer S. (2004). Job stress in national health service managers: a qualitative exploration of the stressor—strain—health relationship. the ‘fit’and ‘unfit’manager. *Int. J. Health Promot. Educ.* 42 48–63. 10.1080/14635240.2004.10708013

[B105] Johnson H. A. M. Spector P. E. (2007). Service with a smile: do emotional intelligence, gender, and autonomy moderate the emotional labor process? *J. Occup. Health Psychol.* 12 319–333. 10.1037/1076-8998.12.4.319 17953492

[B106] Joseph D. L. Newman D. A. (2010). Emotional intelligence: an integrative meta-analysis and cascading model. *J. Appl. Psychol.* 95 54–78. 10.1037/a0017286 20085406

[B107] Kan M. Y. Sullivan O. Gershuny J. (2011). Gender convergence in domestic work: discerning the effects of interactional and institutional barriers from large-scale data. *Sociology* 45 234–251. 10.1177/0038038510394014

[B108] Karatepe O. M. Sokmen A. (2006). The effects of work role and family role variables on psychological and behavioral outcomes of frontline employees. *Tour. Manag.* 27 255–268. 10.1016/j.tourman.2004.10.001

[B109] Kark R. Waismel-Manor R. Shamir B. (2012). Does valuing androgyny and femininity lead to a female advantage? The relationship between gender-role, transformational leadership and identification. *Leadersh. Q.* 23 620–640. 10.1016/j.leaqua.2011.12.012

[B110] Keltner D. Kring A. M. (1998). Emotion, social function, and psychopathology. *Rev. Gen. Psychol.* 2 320–342. 10.1037/1089-2680.2.3.320

[B111] King J. (1995). *Migraine in the Workplace.* Brainwaves: Australian Brain Foundation.

[B112] Koenig A. M. Eagly A. H. Mitchell A. A. Ristikari T. (2011). Are leader stereotypes masculine? A meta-analysis of three research paradigms. *Psychol. Bull.* 137 616–642. 10.1037/a0023557 21639606

[B113] Korabik K. McElwain A. Chappell D. B. (2008). “Integrating gender-related issues into research on work and family,” In *Handbook of Work-Family Integration* eds Whitehead D. L. Korabik K. (Cambridge, MA: Academic Press), 215–232.

[B114] LaFrance M. Banaji M. (1992). Toward a reconsideration of the gender-emotion relationship. *Emot. Soc. Behav.* 14 178–201.

[B115] Lazarus R. S. (1991). Cognition and motivation in emotion. *Am. Psychol.* 46 352–367. 10.1037/0003-066X.46.4.352 2048794

[B116] Lazarus R. S. (1999). The cognition-emotion debate: a bit of history. *Handb. Cogn. Emot.* 5 3–19. 10.1002/0470013494.ch1

[B117] Leka S. Cox T. Zwetsloot G. (2008). *The European Framework for Psychosocial Risk Management. PRIMA-EF: A Resource for Employers and Worker Representatives.* Geneva: World Health Organization.

[B118] Leka S. Griffiths A. Cox T. and World Health Organization (2003). *Work Organisation and Stress: Systematic Problem Approaches for Employers, Managers and Trade Union Representatives.* Geneva: World Health Organization

[B119] Lingard H. Francis V. (2005). Does work–family conflict mediate the relationship between job schedule demands and burnout in male construction professionals and managers?. *Constr. Manag. Econ.* 23 733–745. 10.1080/01446190500040836

[B120] Livingstone H. A. Day A. L. (2005). Comparing the construct and criterion-related validity of ability-based and mixed-model measures of emotional intelligence. *Educ. Psychol. Meas.* 65 757–779. 10.1177/0013164405275663

[B121] Lloyd C. King R. Chenoweth L. (2002). Social work, stress and burnout: a review. *J. Mental Health* 11 255–265. 10.1080/09638230020023642

[B122] Lloyd G. (1984). *The Man of Reason.* Minneapolis: University of Minnesota Press.

[B123] Mark G. (2008). *The Relationship Between Workplace Stress, and Job Characteristics, Individual Differences, and Mental Health.* Ph.D. thesis, Cardiff University, Cardiff

[B124] Martocchio J. J. O’Leary A. M. (1989). Sex differences in occupational stress: a meta-analytic review. *J. Appl. Psychol.* 74 495–501. 10.1037/0021-9010.74.3.495 2737994

[B125] Maslach C. Jackson S. E. (1981). The measurement of experienced burnout. *J. Organ. Behav.* 2 99–113. 10.1002/job.4030020205

[B126] Maslach C. Jackson S. E. Leiter M. P. (1981). *Maslach Burnout Inventory: MBI.* Palo Alto, CA: Consulting psychologists press.

[B127] Maslach C. Jackson S. E. Schwab R.L. (1996). “Maslach burnout inventory - educators survey (MBI-ES),” in *MBI Manual*, 3rd Edn, eds Maslach C. Jackson S.E. Leiter M.P. (Palo Alto, CA: Consulting Psychologists Press).

[B128] Maslach C. Schaufeli W. B. Leiter M. P. (2001). Job burnout. *Annu. Rev. Psychol.* 52 397–422. 10.1146/annurev.psych.52.1.397 11148311

[B129] Mawritz M. B. Greenbaum R. L. Butts M. M. Graham K. A. (2017). I just can’t control myself: a self-regulation perspective on the abuse of deviant employees. *Acad. Manag. J.*, 60 1482–1503. 10.5465/amj.2014.0409

[B130] Mayer J. D. Roberts R. D. Barsade S. G. (2008). Human abilities: Emotional intelligence. *Annu. Rev. Psychol.*, 59 507–536. 10.1146/annurev.psych.59.103006.093646 17937602

[B131] Michael O. Court D. Petal P. (2009). Job stress and organizational commitment among mentoring coordinators. *Int. J. Educ. Manag.* 23 266–288 10.1108/09513540910941766

[B132] Mikolajczak M. Roy E. Luminet O. Fillée C. de Timary P. (2007). The moderating impact of emotional intelligence on free cortisol responses to stress. *Psychoneuroendocrinology* 32 1000–1012. 10.1016/j.psyneuen.2007.07.009 17935898

[B133] Milczarek M. Schneider E. Rial González E. (2009). *OSH in Figures: Stress at Work – Facts and Figures.* Luxembourg: Publications Office of the European Union 10.2802/10463

[B134] Miller A. L. Gouley K. K. Seifer R. Zakriski A. Eguia M. Vergnani M. (2005). Emotion knowledge skills in low-income elementary school children: associations with social status and peer experiences. *Soc. Dev.* 14 637–651. 10.1111/j.1467-9507.2005.00321.x

[B135] Moreno-Jiménez B. Gálvez Herrer M. Rodríguez-Carvajal R. Garrosa Hernández E. (2010). Emotions and health in work settings: analyses of the emotional labour construct and development of a questionnaire. *Rev. Latinoam. Psicol.* 42 63–73.

[B136] Morris M. W. Keltner D. (2000). How emotions work: the social functions of emotional expression in negotiations. *Res. Organ. Behav.* 22 1–50. 10.1016/S0191-3085(00)22002-9

[B137] Morris J. A. Feldman D. C. (1997). Managing emotions in the workplace. *J. Manag. Issues* 9 257–274.

[B138] Mudrack P. E. Farrell G. M. (1995). An examination of functional role behavior and its consequences for individuals in group settings. *Small Group Res.* 26 542–571. 10.1177/1046496495264005

[B139] Neville H. (1998). Workplace accidents: they cost more than you might think. *Ind. Manag.* 40 7–9.

[B140] Nielsen M. B Einarsen S. (2012). Outcomes of exposure to workplace bullying: a meta-analytic review. *Work Stress* 26 309–332. 10.1080/02678373.2012.734709 24885687

[B141] Nolen-Hoeksema S. (1987). Sex differences in unipolar depression: evidence and theory. *Psychol. Bull.* 101 259–282. 10.1037/0033-2909.101.2.259 3562707

[B142] O’Connell C. E. Korabik K. (2000). Sexual harassment: the relationship of personal vulnerability, work context, perpetrator status, and type of harassment to outcomes. *J. Vocat. Behav.* 56 299–329. 10.1006/jvbe.1999.1717

[B143] Oliver S. J. Toner B. B. (1990). The influence of gender role typing on the expression of depressive symptoms. *Sex Roles* 22 775–790. 10.1007/BF00292060

[B144] Osofsky J. D. Osofsky H. J. (1972). Androgyny as a life style. *Fam. Coord.* 21 411–418. 10.2307/582684

[B145] Paoli P. Merllié D. (2001). *Third European Survey on Working Conditions 2000.* Loughlinstown: European Foundation for the improvement of living and working conditions.

[B146] Parsons T. Bales R. F. Family S. (1955). *Interaction Process.* New York, NY: London.

[B147] Patterson M. G. West M. A. Lawthom R. Nickell S. (1997). *Impact of People Management Practices on Business Performance.* London: Institute of Personnel and Development, vii–vii.

[B148] Powell G. N. (1990). One more time: do female and male managers differ?. *Acad. Manag. Perspect.* 4 68–75. 10.5465/ame.1990.4274684

[B149] Powell G. N. Graves L. M. (2003). *Women and Men in Management.* Thousand Oaks: Sage.

[B150] Pugh S. D. (2002). Emotional regulation in individuals and dyads: causes, costs, and consequences. *Emot. Workplace: Underst. Struct. Role Emot. Organ. Behav.* 147:182.

[B151] Purvanova R. K. Muros J. P. (2010). Gender differences in burnout: a meta-analysis. *J. Vocat. Behav.* 77 168–185. 10.1177/1524838018756120 29415630

[B152] Rajgopal T. (2010). Mental well-being at the workplace. *Indian J. Occup. Environ. Med.* 14:63. 10.4103/0019-5278.75691 21461156 PMC3062016

[B153] Ritsner M. Ponizovsky A. Nechamkin Y. Modai I. (2001). Gender differences in psychosocial risk factors for psychological distress among immigrants. *Compr. Psychiatry* 42 151–160. 10.1053/comp.2001.19750 11244152

[B154] Rojo L. M. & Esteban C. G. (2005). “The gender of power: the female style in labour organizations” in *Feminist Critical Discourse Analysis*, ed. Lazar M. M. (London: Palgrave Macmillan), 61–89. 10.1057/9780230599901_3

[B155] Rosenblatt Z. Talmud I. Ruvio A. (1999). A gender-based framework of the experience of job insecurity and its effects on work attitudes. *Eur. J. Work Organ. Psychol.* 8 197–217. 10.1080/135943299398320

[B156] Rospenda K. (2002). Workplace harassment, services utilization, and drinking outcomes. *J. Occup. Health Psychol.* 7:141 10.1037/1076-8998.7.2.141 12003366

[B157] Schein V. E. Mueller R. Lituchy T. Liu J. (1996). Think manager—think male: a global phenomenon?. *J. Organ. Behav.* 17 33–41. 10.1002/(SICI)1099-1379(199601)17:1<33::AID-JOB778>3.0.CO;2-F

[B158] Schmitt M. T. Branscombe N. R. Kobrynowicz D. Owen S. (2002), “Perceiving discrimination against one’s gender group has different implications for well-being in women and men”, *Personal. Soc. Psychol. Bull.* 28 197–210

[B159] Settles I. H. Cortina L. M. Malley J. Stewart A. J. (2006). The climate for women in academic science: the good, the bad, and the changeable. *Psychol. Women Q.* 30 47–58. 10.1111/j.1471-6402.2006.00261.x

[B160] Shepherd D. A. Patzelt H. Wolfe M. (2011). Moving forward from project failure: negative emotions, affective commitment, and learning from the experience. *Acad. Manag. J.* 54 1229–1259. 10.5465/amj.2010.0102

[B161] Shields S. A. (2002). *Speaking from the Heart: Gender and the Social Meaning of Emotion.* Cambridge: Cambridge University Press.

[B162] Shockley K. M. Douek J. Smith C. R. Peter P. Y. Dumani S. French K. A. (2017). Cross-cultural work and family research: a review of the literature. *J. Vocat. Behav.* 101 1–20. 10.1016/j.jvb.2017.04.001

[B163] Smith K. K. Kaminstein D. S. Makadok R. J. (1995). The health of the corporate body: illness and organizational dynamics. *J. Appl. Behav. Sci.* 31 328–351. 10.1177/0021886395313006

[B164] Sojo V. E. Wood R. E. Genat A. E. (2016). Harmful workplace experiences and women’s occupational well-being: a meta-analysis. *Psychol. Women Q.* 40 10–40. 10.1177/0361684315599346 8520964

[B165] Speltz M. L. Bernstein D. A. (1976). Sex differences in fearfulness: verbal report, overt avoidance and demand characteristics. *J. Behav. Ther. Exp. Psychiatry* 7 117–122 10.1016/0005-7916(76)90067-7

[B166] Spence J. T. Buckner C. E. (2000). Instrumental and expressive traits, trait stereotypes, and sexist attitudes: what do they signify?. *Psychol. Women Q.* 24 44–53. 10.1111/j.1471-6402.2000.tb01021.x

[B167] Spence J. T. Helmreich R. L. (1978). *Masculinity and Femininity.* Austin, TX: University of Texas

[B168] Spielberger C. D. Reheiser E. C. (1994). The job stress survey: measuring gender differences in occupational stress. *J. Soc. Behav. Personal.* 9:199.

[B169] Stewart A. J. McDermott C. (2004). Gender in psychology. *Annu. Rev. Psychol.* 55 519–544. 10.1146/annurev.psych.55.090902.141537 14744225

[B170] Tajfel H. (1982). Social psychology of intergroup relations. *Annu. Rev. Psychol.* 33 1–39. 10.1146/annurev.ps.33.020182.000245

[B171] Tajfel H. Turner J. (1986). “The social identity theory of intergroup behaviour,” in *Psychology of Intergroup Relations*, eds Worchel S. Austin W. G. (Chicago: Nelson Hall).

[B172] Tajfel H. E. (1978). *Differentiation Between Social Groups: Studies in the Social Psychology of Intergroup Relations.* Cambridge, MA: Academic Press.

[B173] Tamres L. K. Janicki D. Helgeson V. S. (2002). Sex differences in coping behavior: A meta-analytic review and an examination of relative coping. *Pers. Soc. Psychol. Rev.* 6 2–30. 10.1207/S15327957PSPR0601_1

[B174] Tannen D. (1990). *You Just don’t Understand: Men and Women in Conversation.* New York, NY: Morrow.

[B175] Taylor-Brown S. Johnson K. H. Hunter K. Rockowitz R. J. (1982). Stress identification for social workers in health care: a preventive approach to burn-out. *Soc. Work Health Care* 7 91–100. 10.1300/J010v07n02_07 7344168

[B176] Topa Cantisano G. Moriano León J. A. Morales Domínguez J. (2008). Identidad social y apoyo percibido en las organizaciones: sus efectos sobre las conductas de ciudadanía. *Int. J. Psychol.* 42 363–370

[B177] Turner J.C. (1987). “A self-categorization theory,” in *Rediscovering the Social Group: A Self-Categorization Theory*, eds Turner J. C. Hogg M. A. Oakes P. J. Reicher S. D. Wetherell M. S. (Oxford: Basil Blackwell),42–67

[B178] Verkuil B. Atasayi S. Molendijk M. L. (2015). Workplace bullying and mental health: a meta-analysis on cross-sectional and longitudinal data. *PLoS One* 10:e0135225. 10.1371/journal.pone.0135225 26305785 PMC4549296

[B179] Warr P. (1990). The measurement of well being and other aspects of mental health. *J. Occup. Psychol.* 63 193–210. 10.1111/j.2044-8325.1990.tb00521.x

[B180] Weekes N. Y. Zaidel D. W. Zaidel E. (1995). Effects of sex and sex role attributions on the ear advantage in dichotic listening. *Neuropsychology* 9 62–67. 10.1037/0894-4105.9.1.62

[B181] Weiss H. M. Cropanzano R. (1996). “Affective events theory: a theoretical discussion of the structure, causes and consequences of affective experiences at work”, in *Research in Organizational Behavior: An Annual Series of Analytical Essays and Critical Reviews*, eds Staw B. M. Cummings L. L. (New York, NY: Elsevier Science/JAI Press) Vol. 18 pp. 1–74.

[B182] Wester S. R. Vogel D. L. Pressly P. K. Heesacker M. (2002). Sex differences in emotion: a critical review of the literature and implications for counseling psychology. *Couns. Psychol.* 30 630–652. 10.1177/00100002030004008

[B183] Wharton A. S. (1993). The affective consequences of service work: managing emotions on the job. *Work Occup.* 20 205–232. 10.1177/0730888493020002004

[B184] Whitley B. E. (1983). Sex role orientation and self-esteem: a critical meta-analytic review. *J. Pers. Soc. Psychol.* 44 765–778. 10.1037/0022-3514.44.4.765 6842364

[B185] Williams D. E. D’Alessandro J. D. (1994). A comparison of three measures of androgyny and their relationship to psychological adjustment. *J. Soc. Behav. Personal.* 9:469.

[B186] Wood W. Eagly A. H. (2015). Two traditions of research on gender identity. *Sex Roles*, 73 461–473. 10.1007/s11199-015-0480-2

[B187] Yang S. B. Guy M. E. (2014). Gender effects on emotional labor in Seoul metropolitan area. *Public Pers. Manag.* 44 3–24 10.1177/0091026014550491

[B188] Zaccaro S. J. (2007). Trait-based perspectives of leadership. *Am. Psychol.* 62 6–16. 10.1037/0003-066X.62.1.6 17209675

[B189] Zapf D. Einarsen S. (2005). “Mobbing at work: escalated conflicts in organizations” in *Counterproductive Work Behavior: Investigations of Actors and Targets* eds Fox S. Spector P. E. (Washington, DC: American Psychological Association), 237–270 10.1037/10893-010

[B190] Zastrow C. (1984). Understanding and preventing burn-out. *Br J. Soc. Work* 14 141–155.

