# Corrigendum: Emotional Androgyny: A Preventive Factor of Psychosocial Risks at Work?

**DOI:** 10.3389/fpsyg.2019.00568

**Published:** 2019-03-20

**Authors:** Leire Gartzia, Jon Pizarro, Josune Baniandres

**Affiliations:** ^1^Department of People Management in Organizations, University of Deusto, Bilbao, Spain; ^2^Department of People Management in Organizations, Deusto Business School, Bilbao, Spain

**Keywords:** gender, psychosocial risks, androgyny, emotional competences, work

In the original article, we neglected to include the funder “Generalitat Valenciana” (Spain), “AICO-2017-073” to LG.

The authors apologize for this error and state that this does not change the scientific conclusions of the article in any way. The original article has been updated.

